# Influence of the Mediterranean diet on seminal quality—a systematic review

**DOI:** 10.3389/fnut.2024.1287864

**Published:** 2024-02-15

**Authors:** Clara Ángela Piera-Jordan, Laura Prieto Huecas, Verónica Serrano De La Cruz Delgado, Ana Zaragoza Martí, María Belén García Velert, Cristina Tordera Terrades, Miriam Sánchez-SanSegundo, José Antonio Hurtado-Sánchez, José Tuells, Laura Martín Manchado

**Affiliations:** ^1^Hospital Marina Salud de Denia, Dénia, Spain; ^2^University of Alicante, Alicante, Spain

**Keywords:** semen, quality, diet, Mediterranean, fertility

## Abstract

Currently, there is a growing interest in the study of fertility because fertility-related problems affect up to 15% of the world’s population. The aim of this study was to determine the influence of the Mediterranean diet on seminal quality in men of reproductive age. For this purpose, a systematic review of the literature was carried out following the PRISMA method. Electronic searches were carried out in the international databases PubMed, Scopus, the Cochrane Library, and Web of Science. In total, 10 articles with 2032 men were included. As inclusion criteria, articles published between 2012 and 2022 were selected, including those that included men aged between 18 and 55 years. Nutritional status was assessed through weight, height, and BMI. Dietary habits were evaluated through different indexes and food frequency questionnaires, and finally, semen quality was evaluated by measuring sperm concentration and motility (progressive and non-progressive). In six (60%) of the included articles, a positive relationship between adherence to the Mediterranean diet and semen quality was demonstrated; in two (20%) of the articles, no association was found; and finally, in two (20%) of the included articles, the relationship between dietary patterns typical of DM and semen quality was evaluated. Dietary habits influence semen quality. Adherence to the Mediterranean diet can improve male reproductive health, as it is a diet with antioxidant and anti-inflammatory effects. This is the first systematic review about the influence of the Mediterranean diet on semen quality, and the results are positive. These findings may allow us to provide better advice to our patients and to establish interventions with the aim of improving the results of assisted reproduction techniques.

## 1 Introduction

Research interest in fertility is growing, as approximately 15% of the world’s population—70 million couples of reproductive age—have difficulty conceiving (1–3). The latest estimates from the World Health Organization (WHO), published in 2023, attest to the global nature of this public health problem, as they show little variation in infertility rates across geographic regions or country income groups (4).

The WHO Task Force on the Diagnosis and Treatment of Infertility used standard diagnostic criteria to investigate medical conditions contributing to infertility in 8500 infertile couples from 25 countries around the world (5). Its findings for developed countries identified a female cause in 37% of cases, a male cause in 8%, and a female and male cause in 35%; 5% of couples had an unidentified cause, and 15% became pregnant during the study. By identifying a male cause in 40 to 50% of couples, the study confirmed that infertility was not primarily a female issue. These findings, combined with a growing demand for assisted reproductive technology (ART), have led to growing research into the causes of male infertility (2).

Causes of male infertility can be grouped into four broad categories. The first, accounting for 70 to 80% of cases, is impaired spermatogenesis due to primary testicular dysfunction. Klinefelter syndrome is the main identifiable condition within this category, but most cases of dysfunction have an unknown cause (6). A further 10 to 20% of cases occurring in men with a normal sperm analysis are classified as idiopathic (7). The third category, comprising 5 to 15% of cases, comprises endocrine and systemic disorders presenting with hypogonadotropic hypogonadism (6, 7). The final category (2–5% of cases) is altered sperm motility (7).

To investigate and, where possible, determine the causes of male infertility, it is necessary to perform a thorough history and physical examination according to the WHO Manual for the Standardized Investigation and Diagnosis of the Infertile Couple (8, 9). Semen analysis is the gold-standard laboratory test for investigating male infertility. It can detect low semen volume or a low sperm count (oligozoospermia), reduced motility (asthenozoospermia), and abnormal sperm morphology (teratozoospermia). These alterations can occur in isolation or together (10).

Several studies to date have established that male infertility is influenced by non-modifiable factors such as age and genetics, but modifiable factors including alcohol, tobacco, physical activity, stress, lifestyle, and diet have also been attributed an important role (11–14). Research into environmental factors, including diet, has increased as cumulative exposures have been found to have a major impact on spermatogenesis and male fertility (14).

Over the past years, different dietary patterns, such as the Mediterranean diet, the DASH diet, and the ketogenic diet, have been suggested by researchers for the prevention and management of some chronic diseases (15–17).

The Mediterranean diet is one of the most widely researched diets because of its known contributions to human health. It is recognized worldwide and was declared an Intangible Cultural Heritage of Humanity by UNESCO in 2010 (18). It is based on the eating habits of populations bordering the Mediterranean Sea and consists of fresh, seasonal produce with a high intake of plant-based foods such as fruit and vegetables, legumes, olive oil, nuts, and whole grains; a moderate intake of animal products such as eggs, dairy, poultry, and fish; and a low intake of red and processed meat. It is rich in unsaturated fatty acids, complex carbohydrates, fiber, antioxidants, and anti-inflammatory nutrients (18, 19).

Several systematic reviews and meta-analyses have reported that optimal adherence to the Mediterranean diet may reduce the risk of cardiovascular disease, neurodegenerative disorders such as Alzheimer’s disease, metabolic diseases such as type 2 diabetes mellitus, hypertension, certain types of cancer, and even all-cause mortality (19–23). Most research on the association between diet and male reproductive health has focused on isolated nutrients such as omega 3, selenium, and zinc, with very few studies investigating associations with broader dietary patterns (24). Several cross-sectional studies have found a positive association between adherence to the Mediterranean diet and semen quality, in particular total and progressive sperm motility, which are important factors in male fertility (24–26). A 2.6-increased likelihood of abnormal sperm concentration, total sperm count, and sperm motility has been reported for low compared with high adherence (23). It is believed that the beneficial effects of the Mediterranean diet on semen quality are linked to metabolic factors such as inflammation, oxidative stress, and insulin resistance, which are all related to sperm function (21).

Considering the evidence supporting the positive effects of the Mediterranean diet on male fertility, we designed a systematic review to assess its influence on semen quality in men of reproductive age.

## 2 Materials and methods

We conducted a systematic review following the Preferred Reporting Items for Systematic Reviews and Meta-Analyses (PRISMA) framework (24).

This review has been registered in the PROSPERO database under the code CRD42023424958.

### 2.1 Data sources

Electronic searches were carried out in the international databases PubMed, Scopus, the Cochrane Library, and Web of Science. Additional articles were identified by hand searching the reference lists of the identified articles.

### 2.2 Search strategy

The search strategy was designed to identify full-text, published articles and included MeSH (Medical Subject Heading) terms and the terms *title* and *abstract*. The keywords used, transformed into MeSH terms, were “Mediterranean diet,” “semen,” “quality,” and combined with the Boolean operators AND OR. The search strategy used in PubMed is shown in Table 1.

**Table 1:** Search strategy for PubMed.

Search strategy
#1 (“Mediterranean diet” [Title/Abstract] OR “Mediterranean diet” [MeSH Terms])
#2 (semen [Title/Abstract] OR semen [MeSH Terms])
#3 Quality [Title/Abstract]
#4 2 AND 3
#5 1 AND 4

### 2.3 Article selection

Articles for full-text review were selected by screening the titles and abstracts of all publications retrieved from PubMed, Scopus, the Cochrane Library, and the Web of Science. The articles were independently reviewed by two authors (A.Z.M and C.P.J), who checked the inclusion and exclusion criteria. The quality of each study was also assessed by two authors working separately using the Crombie criteria adapted by Petticrew and Roberts. Any discrepancies were resolved by a third author (L.P.H).

The quality and risk of bias in cross-sectional studies were assessed using the AXIS (26) critical appraisal tool (Table 2). The quality of cohort and case–control studies was assessed using the Newcastle-Ottawa Scale (28) (Table 3). The quality of clinical trials was assessed using the PEDro scale (Table 4). The first and second authors (C.P.J and L.P.H) independently scored each article. Any discrepancies were resolved by agreement with the third author (A.Z.M). Cohen’s kappa statistic (κ) was calculated to assess interrater reliability for risk of bias assessments. Assessment of blinding of participants or observers was not performed, as all the studies were rated as high risk by both authors based on the overall items. Interrater reliability analyzed using Cohen’s κ yielded an intraclass correlation coefficient of 0.8.

**Table 2:** Quality of cross-sectional studies according to the AXIS critical appraisal tool.

Reference	1	2	3	4	5	6	7	8	9	10	11	12	13	14	15	16	17	18	19	20
Cutillas-Tolín et al., 2015 (32)	YES	YES	NO	YES	YES	YES	DK	YES	YES	YES	YES	YES	NO	YES	DK	YES	YES	YES	NO	YES
Karayiannis et al., 2017 (23)	YES	YES	NO	YES	YES	YES	DK	YES	YES	YES	YES	YES	NO	NO	DK	YES	YES	YES	NO	YES
Efrat et al., 2018 (29)	YES	YES	NO	YES	YES	YES	YES	YES	YES	YES	YES	YES	YES	YES	DK	YES	YES	YES	DN	YES
Cutillas-Tolín et al., 2019 (31)	YES	YES	NO	YES	YES	YES	DK	YES	YES	YES	YES	YES	NO	NO	DK	YES	YES	YES	NO	YES
Sala-Huetos et al., 2019 (21)	YES	YES	NO	YES	YES	YES	NO	YES	YES	YES	YES	YES	NO	NO	DK	YES	YES	YES	YES	YES
Ricci et al., 2019 (22)	YES	YES	NO	YES	YES	YES	NO	YES	YES	YES	YES	YES	NO	NO	DK	YES	YES	YES		YES

1. Aims; 2. Study design; 3. Sample size justification; 4. Target reference population; 5. Sampling frame; 6. Sample selection; 7. Non-responders; 8. Measurement validity and reliability; 9. Risk factors and outcomes. 10. Statistics; 11. Overall methods; 12. Basic data; 13. Non-response bias; 14. Non-responders; 15. Internal consistency results; 16. Comprehensive description results; 17. Justified discussions and conclusions; 18. Limitations; 19. Conflict of interest; 20. Ethical approval. DK, Does not know; NR, no reply.

**Table 3:** Quality of cohort and case–control studies according to the Newcastle-Ottawa Scale.

Reference	1	2	3	4	5	6	7	8
Cohort studies								
Salas-Huetos et al., 2022 (33)	a	a	c	a	ab	a	a	A
Case–control studies								
Mendiola et al., 2010 (34)	a	b	a	a	a	b	a	A

1. Representativeness; 2. Selection of non-exposed cohort; 3. Ascertainment of exposure; 4. Outcome; 5. Comparability of cohorts; 6. Assessment of outcome; 7. Follow-up; 8. Adequacy of follow-up. A maximum of one star is allocated for each domain within the “selection” and “outcome” categories; and a maximum of two stars is allocated for “comparability”.

**Table 4:** Quality of randomized clinical trials according to the PEDro scale.

Reference	1	2	3	4	5	6	7	8	9	10	11
Montano et al., 2021 (30)	YES	YES	NO	YES	NO	NO	YES	YES	YES	YES	YES
Salas-Huetos et al., 2018 (27)	NO	YES	YES	YES	YES	YES	YES	YES	YES	YES	YES

1. Eligibility criteria specified; 2. Subjects were randomly allocated to groups; 3. Allocation was concealed; 4. The groups were similar at baseline; 5. There was blinding of all subjects; 6. There was blinding of all therapists who administered the therapy; 7. There was blinding of all assessors who measured at least one key outcome; 8. Measures of at least one key outcome were obtained from more than 85% of the subjects initially allocated to groups; 9. All subjects for whom outcome measures were available received the treatment or control condition as allocated or, where this was not the case, data for at least one key outcome was analyzed by “intention to treat”; 10. Results of between-group comparisons are reported for at least one key outcome; 11. The study provides both point measures and measures of variability for at least one key outcome.

### 2.4 Inclusion and exclusion criteria

The inclusion criteria were (1) open-access articles with an abstract and full text, (2) articles written in English or Spanish, (3) articles published between 2012 and 2022, and (4) articles that included men aged between 18 and 55 years.

The exclusion criteria were (1) articles unrelated to the topic and intervention protocols without results, (2) systematic reviews and meta-analyses, (3) conference proceedings, and (4) studies of karyotype alterations, testicular surgery, testicular cancer, urological disorders, or autoimmune diseases.

### 2.5 Data extraction

The first author extracted all relevant data from the articles, namely, year of publication (2012–2022), study design and objective, year of conduct, sample size, mean participant age, country, study results, and conclusions.

### 2.6 Synthesis of results

The data collected were grouped into two blocks according to (1) the techniques used to assess semen quality, nutritional status, and adherence to the Mediterranean diet and (2) the association detected between the Mediterranean diet and semen quality.

### 2.7 Quality assessment

Study quality was assessed using the Cochrane Collaboration Risk of Bias tool (25), which is a tool developed by the Cochrane Collaboration to determine the quality of evidence in systematic reviews to be reliable for the individual analysis of the included RCTs. This tool consists of seven items covering six domains of bias (bias arising from the randomization process; bias due to deviations from intended interventions; bias due to missing outcome data; bias in the measurement of the outcome; bias in the selection of the reported result; other bias). Each item is classified as having a high, low, or unclear risk of bias.

## 3 Results

**Figure 1:**
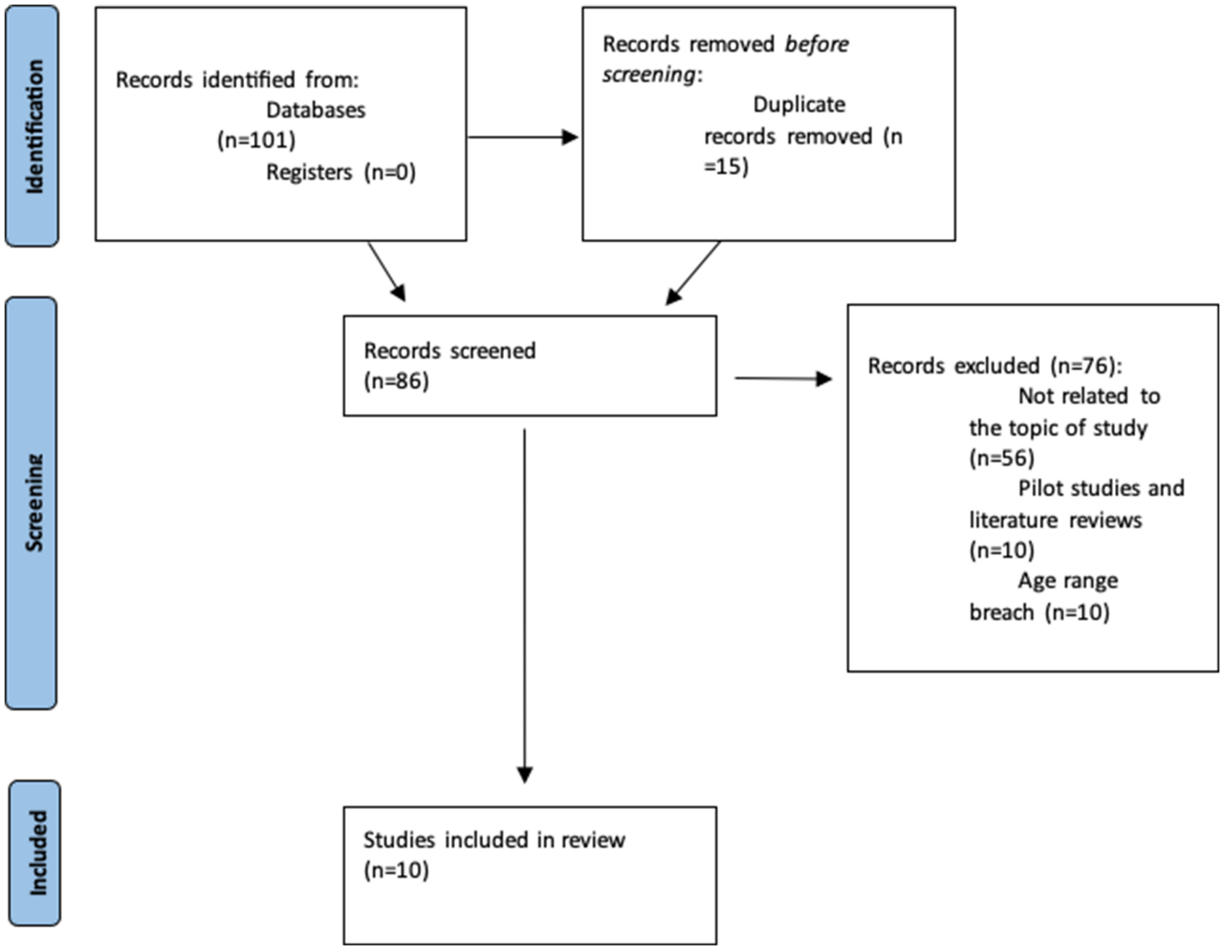
Flow chart showing study selection.

The search yielded 101 articles, 15 of which were duplicates and were immediately removed. Of the 86 remaining articles, 76 were excluded because they met one or more of the exclusion criteria. Ten studies (21–23, 27, 29–34) were thus included in this systematic review (Figure 1).

The characteristics of the 10 articles are summarized in Table 5. Five were conducted in Spain (21, 27, 31, 32, 34), one in Greece (23), one in Israel (29), two in Italy (22, 30), and one in the United States (33). The mean participant age was 28.97 years. There were six cross-sectional studies (21–23, 29, 31, 32), one prospective cohort study (33), one case–control study (34), and two randomized controlled trials (27, 30). The total number of participants was 2032.

**Table 5:** Characteristics of studies included in the analysis.

Authors	Country	Year	Mean age, years	Sample, *n*	Objective	Strengths and limitations	Study design
Cutillas-Tolín et al., 2019 (31)	Spain	2019	20.4	209 men	To determine whether higher adherence to certain dietary indices (rMED, DASH) is associated with improved semen quality and reproductive hormone levels in young men	Limitations: self-reported dietary assessment and time since previous ejaculation. Small sample. Difficult to generalize results because the study population comprised healthy young men with no known fertility problems.	Cross-sectional
Karayiannis et al., 2017 (23)	Greece	2016	38	225 men	To investigate the association between adherence to the Mediterranean diet and semen quality parameters in infertile men using the dietary pattern and the validated Mediterranean Diet Score	Limitations: study design. Difficult to generalize results because the study population comprised men who were in poor health, were overweight, or did not exercise.	Cross-sectional
Sala-Huetos et al., 2018 (27)	Spain	2018	24 in intervention group; 25 in control group	119 men	To assess the effect of chronic consumption of nuts on changes in conventional semen parameters and the potential mechanisms involved	Strengths: study design. Limitations: difficult to generalize results as the study population comprised healthy, apparently fertile men. Sample size. 10% drop-out rate.	Randomized controlled parallel trial
Efrat et al., 2018 (29)	Israel	2018	33.5	280 men	To study the association between semen quality and dietary pattern indices that reflect actual dietary practices and the many combinations by which foods are consumed: HEI, DASH, aMED, and AHEI. (Each index represents a unique combination of dietary components and differs in scoring and classification methodology.)	Strengths: sample size. Use of strict medical and nutritional exclusion and inclusion criteria. Use of dietary pattern analysis instead of nutrient or whole food analysis, which more closely reflects real-world consumption. Limitations: study design. Possible selection bias.	Single-center cross-sectional study
Sala-Huetos et al., 2019 (21)	Spain	2019	24.7	106 men	To investigate associations between adherence to the Mediterranean Diet and semen quality parameters	Strengths: originality of study. Limitations: study design. Results cannot be generalized as population only included healthy, young men. Physical activity was not studied.	Cross-sectional
Montano et al., 2021 (30)	Italy	2021	19.3	263 men	To assess the short-term effects of a diet and physical activity intervention on semen quality in healthy young men living in highly polluted areas of Italy.	Strengths: study design. Limitations: sample size too small to detect significant differences. Effects of intervention only analyzed for 4 months so impossible to determine whether improvements in semen quality would be maintained over time.	Randomized controlled trial
Cutillas-Tolín et al., 2015 (32)	Spain	2015	20.4	215 men	To determine whether dietary patterns are associated with semen quality, reproductive hormone levels, and testicular volume as markers of testicular function.	Limitations: study design. Possibility of residual confounding cannot be ruled out.	Cross-sectional
Sala-Huetos et al., 2022 (33)	United States	2022	36	245 men	To investigate whether men's adherence to eight of the most common healthy dietary patterns is associated with ART outcomes in couples (fertilization, implantation, clinical pregnancy, and live birth) and male semen parameters (ejaculated volume, total sperm count, concentration, motility, and morphology) in the same cohort.	Strengths: prospective design Limitations: diet was only assessed once during follow-up. Possibility of confounding factors not taken into account. Study design.	Prospective cohort
Mendiola et al., 2010 (34)	Spain	2008	34.2 cases, 32.8 controls	61 men	To compare specific nutrient intake between normospermic and oligoasthenoteratospermic patients attending infertility clinics in two Mediterranean provinces of Spain.	Limitations: study design. Sample size	Case-control
Ricci et al., 2019 (22)	Italy	2018	39.3	309 men	To study the association between the Mediterranean diet and abnormal sperm parameters in men of subfertile couples.	Strengths: relatively large sample size. Participants interviewed by same personnel at same clinic. Near-complete participation. Limitations: self-reported dietary information. Accuracy of sperm analysis technique used is debated in the literature. Population limited to infertile men. Possibility of confounding factors not controlled for.	Cross-sectional

AHEI, Alternative Healthy Eating Index; aMED, alternate Mediterranean Diet Score; ART, assisted reproductive technology; DASH, Dietary Approaches to Stop Hypertension; HEI, Healthy Eating Index; rMED, relative Mediterranean Diet Score.

Table 6 summarizes the variables and measurement techniques used to assess nutrition, diet, and semen quality in the different studies.

**Table 6:** Summary of study variables and measurement techniques.

Authors, years; study title	Dietary-nutritional variables	Measurement of dietary-nutritional variables	Semen quality variables	Measurement of semen quality variables
Cutillas-Tolín et al., 2019 (31); Adherence to diet quality indices in relation to semen quality and reproductive hormones in young men	Weight, height, BMI, eating habits, adherence to Mediterranean diet	Digital scale, 101-item FFQ	Concentration, volume, motility (progressive and non-progressive), total sperm count, TMSC, morphology. Male reproductive hormones: FSH, LH, SHBG, testosterone, estradiol, inhibin B, free testosterone. Varicocele (yes/no), and other scrotal abnormalities.	Semen analysis (2010 WHO reference values)
Karayiannis et al., 2017 (23); Association between the Mediterranean diet and semen quality in men in couples attempting fertility	Weight, height, BMI, eating habits, alcohol consumption, adherence to Mediterranean diet	Electronic scale, IPAQ, 75-item FFQ, MDS	Concentration, volume, motility (progressive and non-progressive), total sperm count, morphology, varicocele (yes/no), other scrotal abnormalities.	Semen analysis (2010 WHO reference values)
Sala-Huetos A et al., 2018 (27); Effect of nut consumption on semen quality and functionality in healthy men consuming a Western-style diet: a randomized controlled trial	Weight, height, BMI waist circumference. Eating habits. Glucose, total cholesterol, HDL, LDL, VLDL, insulin, CRP, and folates	Electronic scale, 3-day dietary record, blood tests	Concentration, volume, motility, total count, morphology, pH, vitality, ROS, DNA fragmentation, global DNA methylation, chromosome stability, microRNA expression	Semen samples collected at the beginning of the study and end of the intervention period. Semen analysis (2010 WHO reference values). Chemiluminescence, electron microscopy.
Efrat et al., 2018 (29); Dietary patterns are positively associated with semen quality	Weight, height, BMI, eating habits, and adherence to the Mediterranean diet	Electronic scale, 111-item FFQ	Concentration, volume, motility, total count, morphology	Semen analysis (2010 WHO reference values).

All 10 studies used weight, height, and BMI to evaluate nutrition status (21–23, 27, 29–34). Two also measured waist circumference (27, 30). Eight (21–23, 29–33) evaluated adherence to the Mediterranean diet, while two evaluated dietary habits: one using a 3-day dietary record (27) and the other a 93-item food frequency questionnaire (FFQ) (34).

Several scores were used to measure adherence to the Mediterranean diet. Seven of the eight studies calculated scores based on FFQs validated for the populations being studied (21–23, 29, 31–33), while the other used the more specific PREDIMED [*Prevención con Dieta Mediterránea* (prevention with the Mediterranean diet)] score (30). Two studies used a 101-item FFQ validated in the Spanish population (31, 32). The first, published in 2015, identified two dietary patterns (Mediterranean and Western) based on the frequency with which foods characteristic of the Mediterranean diet were consumed (32). In the second study, published in 2019, the authors used the FFQ to calculate a relative Mediterranean diet score (rMED). Efrat et al. (29) used a 111-item FFQ validated for the Israeli population, while Karayiannis et al. (23) used a 75-item FFQ validated for the Greek population. Salas-Huetos et al. (21) administered a 143-item semi-quantitative FFQ and a 3-day dietary record and subsequently calculated the Trichopoulou Mediterranean Diet (TMD) adherence score. In a later study, Sala-Huetos et al. (33) used information from a validated 131-item semi-quantitative FFQ administered to the same population to calculate several scores, including the TMD score, the alternative Mediterranean diet (AMD) score, and the Panagiotakos Mediterranean diet (PMD) score. Finally, Ricci et al. (22) used a validated 78-item FFQ to calculate the Mediterranean diet score (MDS) in an Italian population.

All 10 studies measured sperm concentration and progressive and non-progressive sperm motility (21–23, 27, 29–34). The reference values used in each case corresponded to fertile men. Nine studies analyzed sperm morphology (21, 23, 27, 29–34), although this parameter had less prognostic significance. The other evaluated reproductive organ diseases (22). Nine studies analyzed sperm volume as a measure of semen quality (21, 22, 26, 27, 29, 31–34). The 10th measured semen total antioxidant capacity (31). Seven studies analyzed total sperm count (21, 23, 27, 29, 31–33), while two analyzed hormones (follicle-stimulating hormone, luteinizing hormone, sex hormone-binding globulin, testosterone, estradiol, inhibin B, and free testosterone) (27, 32). Four studies analyzed the presence of varicocele (22, 23, 31, 32), while two studied semen pH and sperm vitality (21, 27). Just one study calculated the total motile sperm count (31).

Finally, Salas-Huetos et al. (27) evaluated DNA fragmentation, global sperm DNA methylation, chromosome stability, microRNA expression, and reactive oxygen species (ROS), while Cutillas Tolín et al. (32) studied testicular volume.

All the studies used reference values from the WHO Manual for Human Semen Analysis [the 2010 edition in nine cases (21–23, 27, 29–33) and the 1999 edition in one (34)].

Table 7 summarizes the findings of the 10 studies, the associations detected between the Mediterranean diet and semen quality, the reported strengths and limitations, and the quality of the evidence. Six studies reported a positive association between adherence to the Mediterranean diet and semen quality (21–23, 29, 30, 32). Karayiannis et al. (23) found positive effects on sperm concentration, total sperm count, and total and progressive sperm motility (*p* < 0.001). For Salas-Huetos et al. (30), adherence to the Mediterranean diet was positively associated with total (*p* = 0.002) and progressive (*p* = 0.003) sperm motility. Ricci et al. (22) found that lower adherence to the Mediterranean diet was associated with higher odds of low sperm concentration (odds ratio [OR] 1.34; 95% CI, 0.69–2.50 for MDS 4–5 and OR 2.42; 95% CI, 1.21–4.83 for MDS 0–3) compared with high adherence (MDS, 6–9) (*p* = 0.011).

**Table 7:** Association between level of adherence to the Mediterranean diet and semen quality.

Study	Results	Association between Mediterranean diet and semen quality	Strengths and limitations	Level of evidence (GRADE)
Cutillas-Tolín et al., 2019 (31)	Positive associations found between DASH scores and sperm concentration (*P* = 0.004), total sperm count (*p* = 0.004), and total sperm motility (*P* = 0.002).	No association found between adherence to Mediterranean diet and semen quality.	Limitations: dietary evaluation and time since previous ejaculation were self-reported. Small sample. Difficult to generalize results due to the type of sample.	Very low ⊕⊝⊝⊝
Karayiannis et al., 2017 (23)	Abnormal semen analysis parameters associated with higher BMI (25.6 vs. 24, *p* = 0.021) and lower mean MDS (33 vs. 35, *p* = 0.002). MDS positively associated with sperm concentration, total sperm count, total and progressive sperm motility (*P* < 0.001), and normal sperm morphology (%) (*p* = 0.025)	Higher adherence to the Mediterranean diet may help improve semen quality, but this does not necessarily mean improved male fertility.	Limitations: study design. Difficult to generalize results due to the type of sample.	Low ⊕ ⊕ ⊝⊝
Sala-Huetos et al., 2018 (27)	In the intervention group (inclusion of nuts in diet) significant improvement in: total sperm count (*P* = 0.002), sperm vitality (*P* = 0.003), total sperm motility (*p* = 0.006), progressive sperm motility (*p* = 0.036), and sperm morphology (*p* = 0.008). Significant decrease in sperm DNA fragmentation (*p* < 0.001) that would explain previous findings.	Chronic consumption of nuts within the Mediterranean diet improves total sperm count, vitality, motility, and morphology.	Strengths: study design. Limitations: results difficult to generalize to the general population. Sample size. 10% drop-out rate.	Moderate ⊕⊕⊕⊝

Two studies found no association between adherence to the Mediterranean diet and semen quality or ART success (31, 33), but one of them (31) did find a positive association between Dietary Approaches to Stop Hypertension (DASH) scores and sperm concentration (*p* = 0.004), total sperm count (*p* = 0.004), and total sperm motility (*p* = 0.002). Finally, two studies reported a positive association between typical Mediterranean dietary patterns and semen quality (27, 34).

The most common limitations mentioned were sample size, low generalizability, and study design (most of the studies were observational). The quality of evidence was assessed using the Grading of Recommendations, Assessment, Development, and Evaluation (GRADE) system, which is an important tool in evidence-based medicine approaches (35). All the studies had a very low, low, or moderate level of evidence, as they were largely observational and contained some bias (Table 7). The risk of bias for the two randomized controlled trials was analyzed using Cochrane RoB2 traffic light plots (36) (Table 8). One of the trials had a low risk of bias (27), while the other generated some concerns about bias related to deviations from the intended intervention and evaluation of results (30).

**Table 8:** Risk of bias in randomized controlled trials.

		Risk of bias domains					
		D1	D2	D3	D4	D5	Overall
Study	Montano et al., 2021 (30)	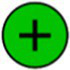	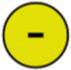	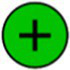	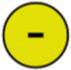	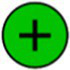	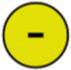
	Salas-Huetos et al., 2018 (27)	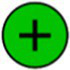	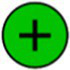	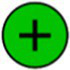	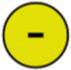	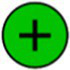	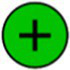

Domains: D1: Bias arising from the randomization process. D2: Bias due to deviations from intended intervention inline figure some concerns. D3: Bias due to missing outcome data. D4: Bias in measurement of the outcome. Inline figure Low. D5: Bias in the selection of the reported result. Judgment: 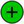 some concerns; 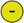 Low.

Six studies showed an association between high adherence to the Mediterranean diet and semen quality (21–23, 29, 30, 32) (Table 9). Three of them reported that men with high adherence levels had significantly better semen quality (23, 29, 30). Salas-Huetos et al. (21) showed a significant association between adherence and semen motility, while Cutillas-Tólin et al. (32) reported that the diet was positively associated with total sperm count.

**Table 9:** Results according to the effect of adherence to the Mediterranean diet on semen quality.

Authors, years	↑ Adherence to the Mediterranean diet	↓ Adherence to the Mediterranean diet
	Semen quality	Semen quality
Cutillas-Tolín et al., 2019 (31)	-	-
Karayiannis et al., 2017 (23)	↑	
Sala-Huetos et al., 2018 (27)	-	-
Efrat et al., 2018 (29)	↑	
Sala-Huetos et al., 2019 (21)	↑ (motility)	
Montano et al., 2021 (30)	↑	
Cutillas-Tolín et al., 2015 (32)	↑ (total sperm count)	
Sala-Huetos et al., 2022 (33)	-	-
Mendiola et al., 2010 (34)	-	-
Ricci et al., 2019 (22)	↑	

↑ = improvement, ↓ = decrease; −: no association.

## 4 Discussion

The aim of this systematic review was to evaluate the association between adherence to the Mediterranean diet and semen quality in men of reproductive age. Of the 10 studies analyzed, 6 showed a positive influence on semen parameters (21–23, 29, 30, 32). Two studies did not find a significant association between the Mediterranean diet and either semen quality or ART success (31, 33), while the other two found that certain characteristics of the Mediterranean diet (e.g., consumption of foods rich in antioxidants or dried fruit such as walnuts) improved semen parameters (27, 34). The most widely used variables used to assess nutritional status were weight, height, and BMI. rMED and TMD, calculated from validated FFQs, were the main scores used to assess dietary habits. The most common parameters used to assess semen quality were sperm concentration, volume, and motility (some studies also analyzed sperm morphology).

In close agreement with previous research, the findings of this systemic review show that adherence to the Mediterranean diet has beneficial effects on semen quality (21–23, 27, 29, 30, 32–34). Eslamian et al. (37) found that the intake of different food groups present in the Mediterranean diet (fruit, vegetables, chicken, skimmed milk, and seafood) significantly reduced the risk of asthenozoospermia. Braga et al. (38) concluded that cereal and fruit consumption had respective benefits for sperm concentration and motility. Toxins such as tobacco and alcohol (38, 39), by contrast, had negative effects, as did a high BMI (38). In a meta-analysis of six cross-sectional studies involving 708 men aged between 18 and 60 years (four of the studies evaluated the Mediterranean diet), Lei-Lei Cao et al. (40) concluded that healthy dietary patterns similar to Mediterranean patterns (high vegetable content and low saturated fat content) had beneficial effects on sperm concentration, total sperm count, and progressive motility. Based on their findings, they recommended that clinical practitioners promote healthy eating habits as a means of improving semen quality. In a study of 254 cases and 633 controls, Cui et al. (41) observed that higher scores on the Chinese Health Eating Index (CHEI), the Alternate Healthy Eating Index (AHEI), and DASH were associated with reduced asthenozoospermia risk. CHEI is used to evaluate diet quality according to the Dietary Guidelines for Chinese people, which recommend a high intake of cereal (especially whole grains), tubers, legumes, fruit, dairy products, soybeans, fish and shellfish, poultry, eggs, seeds, and nuts, and a low intake of red meat, cooking oil, sodium, added sugars, and alcohol. Finally, a Cochrane meta-analysis of couples seeking fertility treatment observed a slight increase in live birth rates when subfertile men received oral supplementation with antioxidants, which occur naturally in many foods in the Mediterranean diet. The finding, however, was based on low-quality evidence (33).

Not all studies have found beneficial effects for the Mediterranean diet. Sala-Huetos et al. (33), for example, found no association between adherence to this diet and semen parameters. They actually found inverse associations between higher adherence and lower fertilization rates, but suggested that these were perhaps chance findings as they had no effect on the main clinical outcomes. Discrepancies in findings could be related to differences in the nature of the dietary analyses, with some studies analyzing isolated food products and nutrients and others diet and lifestyle patterns.

The beneficial effects of the Mediterranean diet on semen quality are linked to the intake of different nutrients found in this diet, which is rich in long-chain polyunsaturated fatty acids—essential for sperm membrane integrity (42–44) and an important component of seminal plasma—and low in trans and saturated fatty acids, which have been linked to lower sperm quality, particularly in terms of concentration, motility, and morphology (37, 43–45). The Mediterranean diet is also rich in antioxidants, which counteract the effects of what is considered to be one of the main causes of idiopathic infertility: oxidative stress (46). Oxidative stress affects sperm in a number of ways: it alters protein synthesis and membrane integrity (reducing motility and fertilization ability), increases DNA fragmentation (affecting genetic integrity) (23, 46–55), and creates an inflammatory environment. All these factors are detrimental to male reproductive health (41).

Finally, the Mediterranean diet is characterized by a high intake of anti-inflammatory nutrients and a low intake of proinflammatory nutrients (47), helping to protect against anatomic or functional alterations to sperm (56).

This systematic review has several limitations. First, we may have missed some evidence as we limited our search to English- and Spanish-language articles published in PubMed, Scopus, the Cochrane Library, and the Web of Science. The tools used to evaluate adherence to the Mediterranean diet were highly heterogeneous, as there is no single internationally validated tool for this purpose. This variability could affect the external validity of our findings. By excluding studies of patients who had undergone ART, we considerably reduced the number of studies eligible for inclusion. Finally, just two of the studies were randomized controlled trials; the rest were observational studies. The main strength of this systematic review is its design, as integrating results from multiple studies offers greater statistical power and external validity. We also used standardized critical appraisal tools to assess the quality of the studies included. Finally, our review provides information that can be applied in clinical practice.

It is the first study to systematically analyze the association (found to be positive) between the Mediterranean diet and semen quality.

Most studies to date have examined the influence of specific micronutrients on semen parameters (27, 31, 34, 37, 42–45, 57–60), but it would seem more appropriate to analyze dietary patterns, as different nutrients and food products can have synergistic or antagonistic interactions (61). A more holistic approach would probably improve our understanding of the overall impact of the Mediterranean diet on semen quality. This review is thus clinically relevant and contributes significantly to the research in this field.

## 5 Conclusion

Dietary habits can influence semen quality in men of reproductive age. Adherence to healthy diets such as the Mediterranean diet has beneficial effects on male reproductive health, with reports of better semen parameters in relation to the intake of foods rich in polyunsaturated fatty acids, antioxidants, and anti-inflammatory substances, which contribute to reducing oxidative stress and protecting against its negative effects on sperm. Healthy eating habits may also protect against infertility, suggesting a need for dietary counseling in couples planning a pregnancy or undergoing ART.

Further investigation on the links between diet and semen quality is needed to inform health promotion and disease prevention strategies focused on improving the dietary habits of men of reproductive age.

## Data availability statement

The original contributions presented in the study are included in the article/supplementary material, further inquiries can be directed to the corresponding author.

## Author contributions

CP-J: Conceptualization, Data curation, Investigation, Methodology, Project administration, Resources, Visualization, Writing – original draft, Writing – review & editing. LP: Conceptualization, Formal analysis, Investigation, Methodology, Validation, Visualization, Writing – original draft. VS: Writing – original draft, Writing – review & editing, Conceptualization, Data curation, Formal analysis, Investigation, Supervision, Validation. AZ: Conceptualization, Formal analysis, Methodology, Project administration, Software, Supervision, Writing – review & editing. MG: Conceptualization, Investigation, Methodology, Validation, Visualization, Writing – original draft. CT: Project administration, Resources, Validation, Visualization, Writing – original draft. MS-S: Conceptualization, Investigation, Supervision, Writing – review & editing. JH-S: Funding acquisition, Project administration, Supervision, Writing – review & editing. JT: Conceptualization, Funding acquisition, Project administration, Supervision, Writing – review & editing. LM: Conceptualization, Data curation, Investigation, Methodology, Resources, Validation, Writing – original draft, Writing – review & editing.
